# Guideline for designing microbiome studies in neoplastic diseases

**DOI:** 10.1007/s11357-024-01255-4

**Published:** 2024-06-26

**Authors:** Edit Mikó, Adrienn Sipos, Emese Tóth, Andrea Lehoczki, Monika Fekete, Éva Sebő, Gábor Kardos, Péter Bai

**Affiliations:** 1 Department of Medical Chemistry, Faculty of Medicine, University of Debrecen, Egyetem Tér 1., 4032 Debrecen, Hungary; 2 HUN-REN-DE Cell Biology and Signaling Research Group, 4032 Debrecen, Hungary; 3 Department of Hematology and Stem Cell Transplantation, South Pest Central Hospital-National Institute for Hematology and Infectious Diseases, Budapest, Hungary; 4 Doctoral College, Health Sciences Program, Semmelweis University, Budapest, Hungary; 5 Department of Public Health, Semmelweis University, Budapest, Hungary; 6 Breast Center, Kenézy Gyula Hospital, University of Debrecen, 4032 Debrecen, Hungary; 7 Department of Metagenomics, University of Debrecen, 4032 Debrecen, Hungary; 8 Faculty of Health Sciences, One Health Institute, University of Debrecen, 4032 Debrecen, Hungary; 9 MTA-DE Lendület Laboratory of Cellular Metabolism, 4032 Debrecen, Hungary; 10 Research Center for Molecular Medicine, Faculty of Medicine, University of Debrecen, 4032 Debrecen, Hungary; 11 Center of Excellence, The Hungarian Academy of Sciences, Budapest, Hungary

**Keywords:** Cancer, Microbiome, Guideline, Stage, Grade, Molecular subtype, Aging, Age

## Abstract

Oncobiosis has emerged as a key contributor to the development, and modulator of the treatment efficacy of cancer. Hereby, we review the modalities through which the oncobiome can support the progression of tumors, and the emerging therapeutic opportunities they present. The review highlights the inherent challenges and limitations faced in sampling and accurately characterizing oncobiome. Additionally, the review underscores the critical need for the standardization of microbial analysis techniques and the consistent reporting of microbiome data. We provide a suggested metadata set that should accompany microbiome datasets from oncological settings so that studies remain comparable and decipherable.

## Introduction

Cancer remains one of the leading causes of morbidity and mortality worldwide, with breast cancer accounting for a significant proportion of these cases [1, 2]. Amidst the myriad of factors contributing to cancer progression, the role of microorganisms, particularly bacteria, has emerged as a critical area of research [3–10]. Oncobiome refers to a microbiome with altered composition in cancer patients, which is implicated in supporting progression, and metastasis of various cancers, including breast cancer [11–13]. The oncobiome can influence cancer through direct interactions with cancer cells, modulation of the immune system, or alterations in the local tumor microenvironment [13]. The oncobiome is thought to interact with anticancer therapy as well, influencing efficacy and outcome [14]. This review aims to dissect the current understanding oncobiosis, highlighting key findings, challenges, and future directions in this evolving field and to provide a comprehensive reporting and sampling guideline for oncobiome studies.

## ncobiome: an overview

The intricate relationship between the human microbiome and cancer has garnered significant attention, leading to the identification of a phenomenon known as oncobiosis [15]. Oncobiosis refers to the dysbiosis of microbiome compartments in the presence of neoplasia, which is often associated with cancer progression, representing a shift away from the normal microbial balance [15]. This alteration in the microbial ecosystem is not restricted to a single niche but affects multiple microbiome compartments that for example in breast cancer includes, but is likely not limited to, the fecal/gut, breast tissue, milk ducts, tumor sites, oral cavity, pharyngeal region, urinary tract, and even the blood (Table 1, [3]). It is important to note that the specific pattern and impact of oncobiotic changes vary across different types of cancer, underscoring the complex and distinct nature of microbial associations with neoplastic diseases [102, 103].

**Table 1:**
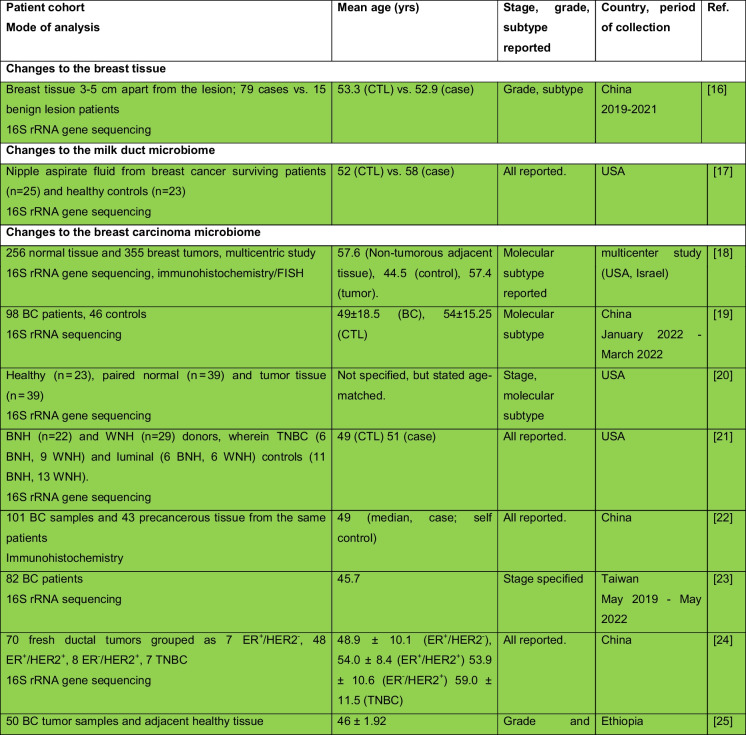

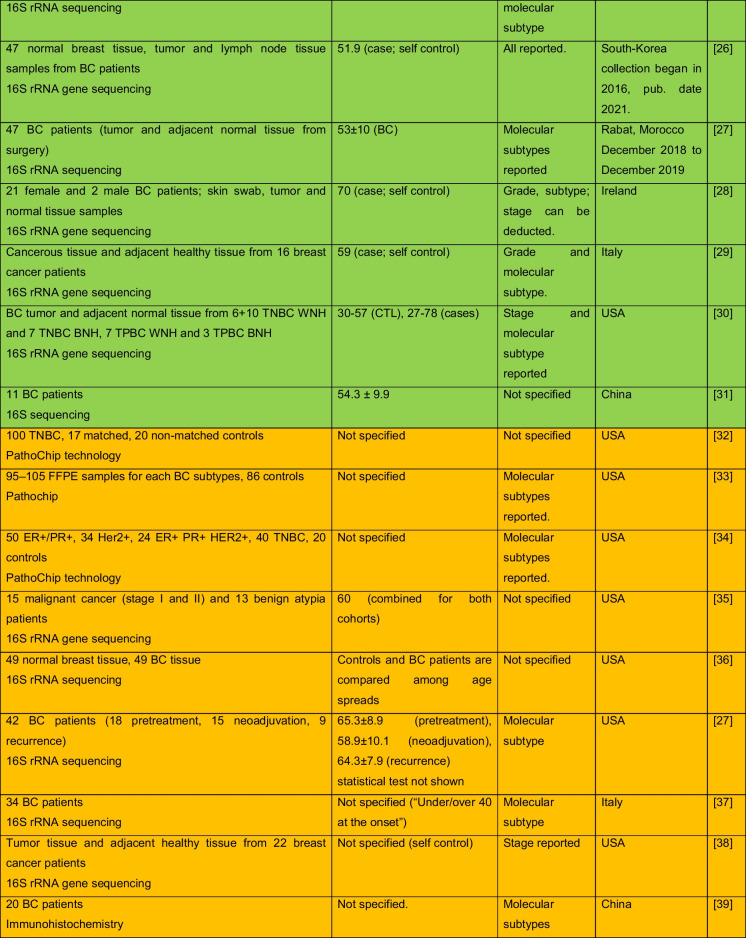

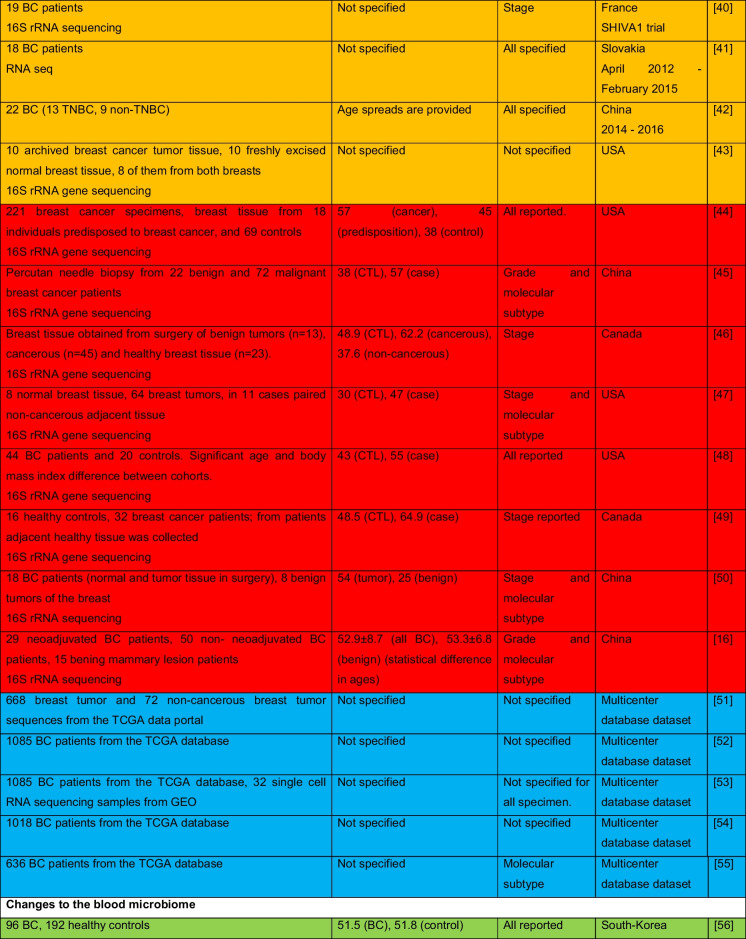

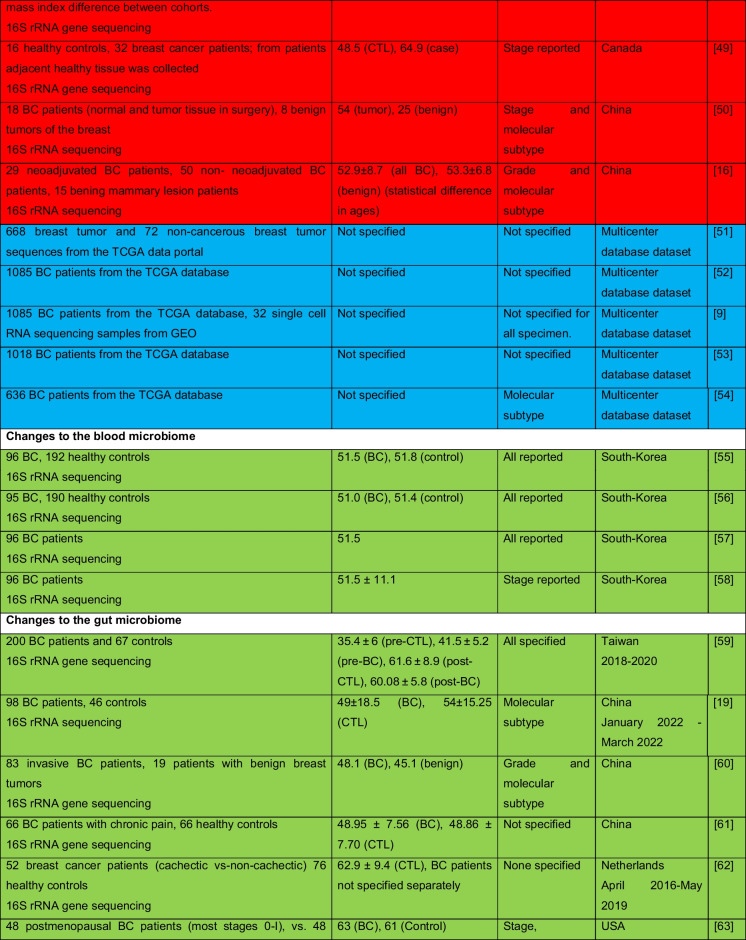

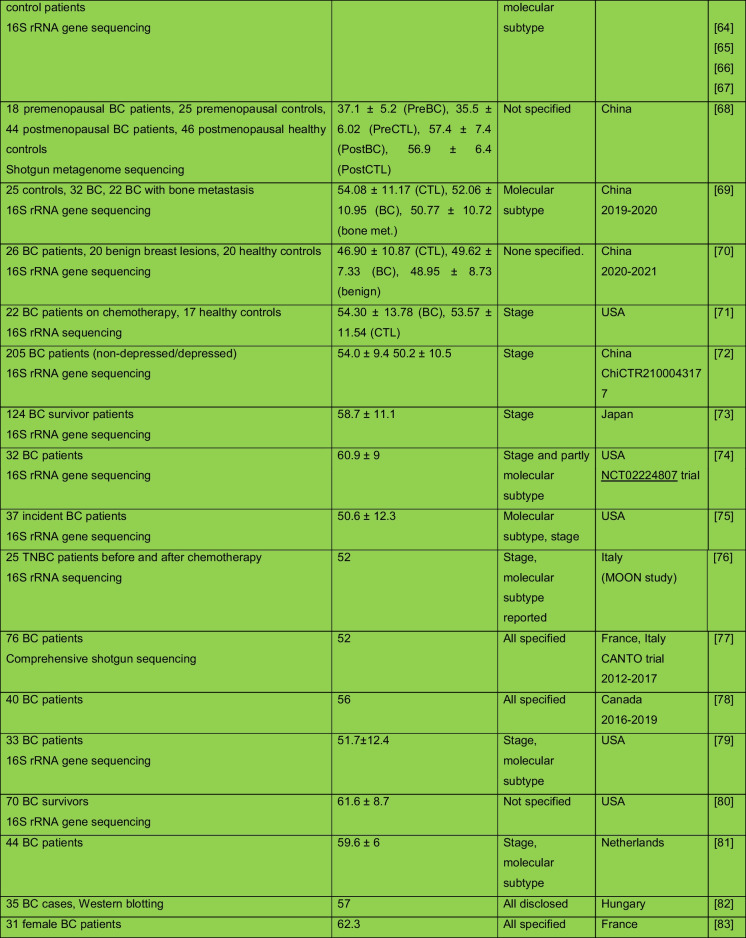

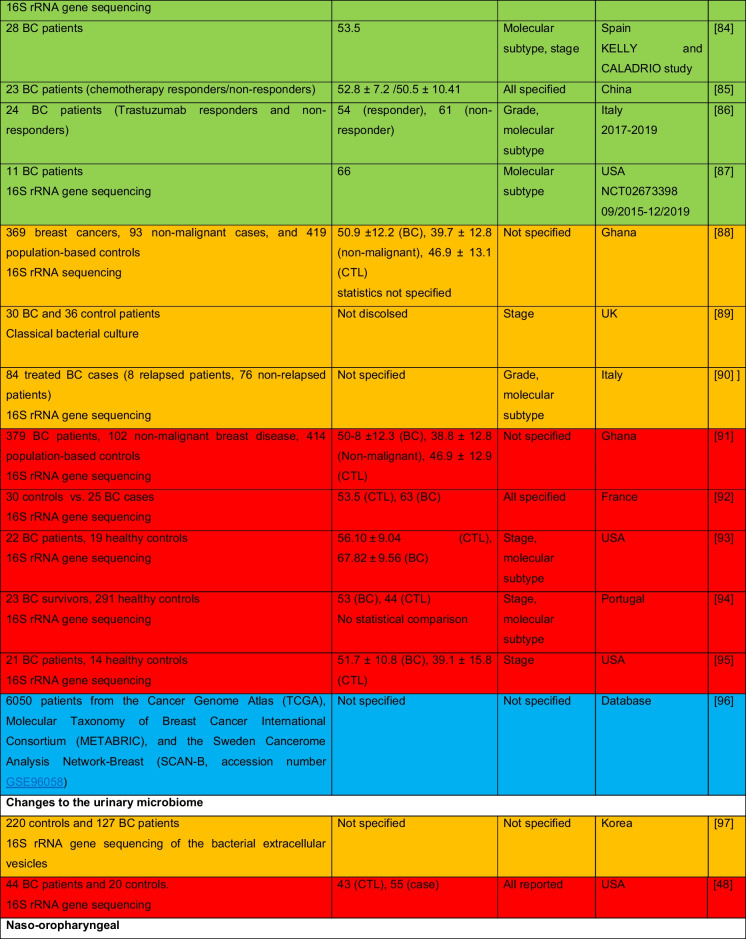

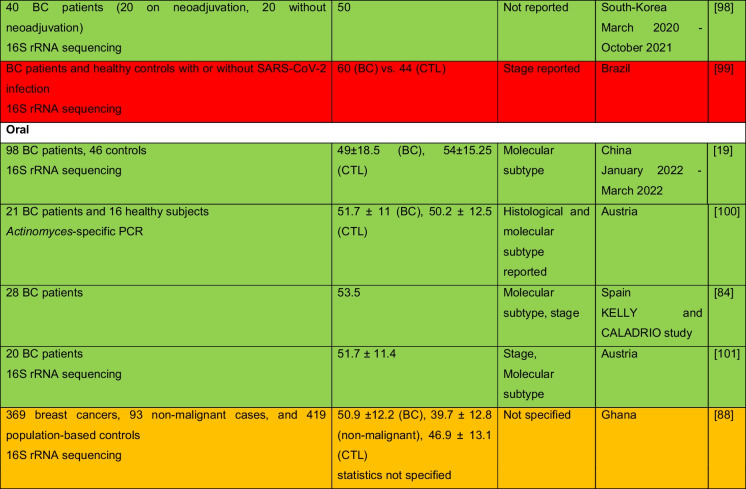
Changes to the gut microbiome in breast cancer

Abbreviations: *BC* breast cancer, *BNH* African American as Black non-Hispanic, *CTL* control, *ER* estrogen receptor, *FISH* fluorescence in situ hybridization, *HER2* epidermal growth factor receptor 2, *N/A* not applicable, *TNBC* triple-negative breast cancer, *WNH* Caucasian as White non-Hispanic

Collection dates were specified only if those were specified in the manuscript

Green: cohorts are age-matched or self-control study

Orange: does not report age

Red: cohorts are not age matched

Blue: age criteria not applicable, e.g., data reanalysis, database mining

While the list of bacteria directly implicated in oncogenesis remains relatively concise, the broader concept of oncobiosis encompasses a range of microbial interactions that support cancer progression and influence disease outcomes [104]. These interactions manifest through three, somewhat overlapping primary modalities:

1. Colonization of tumor tissue: Certain bacteria have been found to colonize tumor tissues, triggering chronic inflammation and/or secreting toxins that can promote tumor growth and progression or induce genomic instability [3, 13, 46]. Bacteria in the oncobiome can suppress the activity of immune cells capable of targeting cancer cells, such as natural killer (NK) cells and T-cells. The specific mechanisms of immune modulation can include the alteration of cytokine and chemokine profiles, modification of antigen presentation, and the induction of immunosuppressive cells within the tumor microenvironment [3, 13, 46].
2. Production of metabolites and toxins with paracrine or hormone-like properties: Microbial metabolites and toxins can act in a paracrine fashion or mimic hormone action, influencing cancer cell behavior and tumor microenvironment dynamics [3, 105, 106].
3. Immune modulation/immune evasion: The microbiome plays a crucial role in modulating the host immune response, which can influence the efficacy of immune surveillance against tumors and impact cancer progression [3, 64, 107, 108].

Significantly, the microbiome has been identified as a pivotal player in controlling metastasis formation, thereby determining the overall outcome of the disease [31, 40, 108, 109]. This suggests that interventions targeting oncobiotic changes could potentially offer novel therapeutic avenues for cancer treatment and metastasis prevention.

A handful of bacteria have been firmly established in the literature as being linked to cancer as tumor inducers. *Helicobacter pylori* is well-known for its association with gastric cancer [110–112] and mucosa-associated lymphoid tissue lymphoma [113–116]. Several bacterial species play significant roles in the initiation and progression of colorectal cancer through the induction of chronic inflammation, production of genotoxic substances, and modulation of the immune response [117–119]. *Fusobacterium nucleatum* can adhere to and invade colorectal epithelial cells using its FadA adhesin to bind to E-cadherin declutching β-catenin signaling [120–122], promotes a pro-inflammatory environment [123–126] and interacts with the host’s immune system to shield tumor cells from immune surveillance [127–132] conducive to cancer progression. Enterotoxigenic *Bacteroides fragilis* (ETBF) secretes a toxin known as *Bacteroides fragilis* toxin (BFT) that can disrupt the mucosal barrier and can induce oncogenic signaling pathways as STAT3 and NF-κB, and contribute to the formation of a tumorigenic environment in the colon [131, 133–136]. Strains of *Escherichia coli* (strains harboring the polyketide synthase gene, pks +) produce colibactin, a genotoxin that causes DNA damage and mutations, thereby contributing to colorectal carcinogenesis [137–139]. *Streptococcus gallolyticus* (formerly known as *Streptococcus bovis* biotype I) has been associated with an increased risk of colorectal cancer [140–145] through inducing chronic inflammation and potentially modulating the immune response within the gastrointestinal tract. 

The number of directly oncogenic bacteria, as mentioned, is only a handful, and these species are not necessarily overrepresented in neoplasia patients. Oncobiosis in these cases yields a maladaptive microbiome with disproportionate, abnormal taxonomical composition coinciding with pathological functional adaptation with large interpersonal variability.

## Detection and analysis of the oncobiome

The detection and analysis of oncobiome are crucial for understanding the microbiome’s role in cancer development and for exploring new diagnostic and therapeutic strategies. This section covers the contemporary techniques and methodologies employed in assessing the oncobiome, highlighting the challenges and limitations of these methods.

### 16S rRNA gene sequencing

16S rRNA gene sequencing targets the hypervariable regions of the 16S ribosomal RNA gene that can distinguish between different taxa. The advantage of the 16S hypervariable region sequencing is that it can provide taxonomical information from samples with low bacteria-to-host nucleic acid ratio and its relative cheapness compared to whole genome sequencing or to shotgun sequencing (see below). However, despite its widespread use and valuable insights, 16S rRNA gene sequencing comes with important limitations, particularly concerning resolution at the genus and species level. The method relies on the analysis of specific hypervariable regions within the 16S rRNA gene to distinguish between bacterial taxa. However, the degree of variability in these regions can differ among taxa; therefore, the resolution may not be sufficient to distinguish closely related genera in some cases [146]. The challenge becomes even more pronounced at the species level, where the variability in the 16S rRNA gene may not be sufficient to distinguish between closely related species. This is particularly problematic in the context of oncobiome, where different species within the same genus may have vastly different (or even opposite) effects on cancer development. Another technical issue is that 16S rRNA sequencing may skew the representation of low-abundance taxons as compared to whole genome sequencing [147]. It is possible to predict functional changes of microbial communities using algorithms as PICRUSt [148]. It is also important to note that samples with low abundance of bacterial DNA are prone for pollution from the environment (e.g., the skin in samples from surgical interventions, pollution of reagents, or pollution of tap water from formalin-fixes, paraffin-embedded blocks, or even from plasticware and kits [149]) that needs to be meticulously controlled.

### Whole genome sequencing (WGS)

Shotgun sequencing is a powerful tool that revolutionized our understanding of the microbiome’s complexity and its impact on health and disease. By sequencing all the DNA (metagenomics) or RNA (metatranscriptomics) in a sample, these methods allow for the identification of not only bacterial communities, but also viruses, fungi, and other microorganisms, offering a comprehensive view of the microbial ecosystem. In addition, metagenomics and metatranscriptomics provide insights into the genetic and functional potential of these microbial communities.

Despite their considerable advantages, metagenomics and metatranscriptomics come with limitations. One such limitation is cost. Further, the large volume of data generated requires substantial computational resources for storage, processing, and analysis. Moreover, data analysis is complex and challenging; it requires sophisticated bioinformatic tools and expertise to assemble, annotate, and interpret the taxonomical composition and biochemical functions of a bacterial community. Another limitation is the signal-to-noise ratio in samples with low microbial biomass, such as human tissue. The DNA from the host or other sources (e.g., environmental contaminants) can overwhelm the microbial DNA, making it challenging to accurately profile the microbiome. Tackling this requires careful sample handling, processing, and data analysis techniques to minimize contamination and to ensure that the microbial signal can be accurately detected and analyzed as well as the use of environmental controls.

Metagenomics and metatranscriptomics of human-associated samples raise ethical and privacy concerns, particularly when human DNA is sequenced alongside microbial DNA. The potential for identifying genetic information about the host requires careful consideration of consent, data storage, and data sharing practices to protect individuals’ privacy.

### Hybridization-based techniques

The PathoChip or the GeoChip technologies are based on the hybridization of nucleic acid samples from biomaterials to probes attached to the chip [150, 151]. The probes on the GeoChip surface recognize bacteria, archaea, fungi, protists, and viruses [150]. In addition, the PathoChip contains probes for virulence factors, toxin, and siderophore genes; hence, the system provides besides taxonomical, microbial function information [151]. This methodology is an alternative for low bacterial-to-host nucleic acid ratio samples. The technology has similar advantages and drawbacks as the hybridization-based transcriptomic and genomic experimental techniques [152]. Multiple studies were conducted using the technology on multiple neoplasias [34, 153–156].

### Quantitative polymerase chain reaction (qPCR)

qPCR is employed to quantify specific bacterial species or genes of interest in tissue samples. This technique is valuable for confirming the presence of a specific bacterial species or a bacterial gene identified through sequencing methods.

### Imaging techniques

Fluorescence in situ hybridization (FISH) and immunohistochemistry (IHC) are used to visualize bacteria within tissue samples, allowing researchers to examine the spatial distribution of the oncobiome in relation to cancer cells and the tumor microenvironment (as an example see [18]).

### Classical bacterial culture techniques

Culturable bacteria were isolated from human tumors [18]. This observation points towards the possible applicability of classical bacterial culture in characterizing bacterial communities (hereby we cite an example of a non-neoplastic disease [157]). The advantage of this approach is that the bacteria can be actually characterized in terms of immunogenic and biochemical properties or their effects on cancer (e.g., can be fed to an animal with cancer). The flip side of this approach is that unculturable bacteria cannot be assessed.

### Optimizing oncobiome research in cancer: guidelines and standards

The incidence of neoplastic diseases escalates with advancing age [158], a phenomenon paralleled by aging-related shifts in the microbiome [159–167]. These microbiome changes are implicated in a range of aging-associated conditions, from cognitive decline to systemic inflammation and metabolic diseases linked to aging [168, 169]. Moreover, aging influences the microbiome’s composition, notably diminishing its diversity across various compartments [159–167]. This observation hints at a potential connection between aging-associated dysbiosis and heightened neoplastic disease risk in the elderly.

Amidst the surge in microbiome-related studies, research on oncobiosis has been expanding at a similar pace. However, not all studies meet the high standards required for impactful scientific contributions, as highlighted in select critiques [170]. This discrepancy underscores the urgent need for standardized guidelines to aid in the structuring of microbiome-related oncological research. Our objective is to contribute towards the standardization of patient cohort characterization, using breast cancer-related studies as a benchmark. Recent general guidelines for microbiome research have been set forth by Mirzayi et al. [171] and Bharti et al. [172], providing a foundational technical checklist.

In reviewing the literature on microbiome studies related to breast cancer (Table 1)—chosen as a model due to the correlation between breast cancer risk and age [173]—we screened PubMed using “microbiome” and “breast cancer” as search terms. Our analysis covered 90 papers focusing on human breast cancer, with the majority examining changes in the tumor tissue microbiome (39) and the GI tract microbiome (36). A smaller fraction addressed other compartments. Notably, approximately 40% of all studies, and a worrying 55% of cross-sectional studies, neglected to report participant ages or ensure age-matched cohorts, failing to account for age-related microbiome composition shifts.

**Fig. 1:**
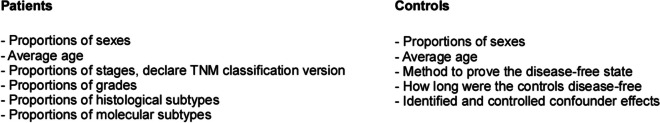
A recommended panel of information to be disclosed on the study subjects in microbiome studies on neoplasia patients

The consistency in clinical characterization of study participants often falls short, hampering the comparability of research findings. Common characterization criteria include cancer stage, grade, and molecular subtype [174–178], with reporting frequencies of 57%, 34%, and 71%, respectively. Molecular subtype classification typically hinges on the expression profiles of the estrogen receptor, progesterone receptor, and HER2 receptor. However, detailed reporting on luminal A and B subtypes separately is rare. When documenting these variables, specifying the version of the classification system used is crucial. These details should accompany the sequencing data as metadata (refer to Fig. 1 for a recommended panel of patient data). For stratified cohorts, reporting should extend to sex distribution, average age among subgroups, and, for non-incident patients, medication and chemoradiotherapy regimens.

Control group selection criteria, including disease-free status assessment and duration of being disease-free (i.e., were the controls checked once before sampling for being disease-free or is the disease-free status maintained over extended periods), should be transparent. Furthermore, the study should account for potential confounders such as smoking habits, nutrition, BMI, menarche, and ECOG status. Sampling conditions, including the time lapse between sample collection and biobanking and the use of nucleic acid preservatives, must also be disclosed. Power calculations are advisable for experimental cohort setup, enhancing study robustness and reliability.

By adhering to these comprehensive guidelines, the microbiome research community can ensure greater consistency and comparability across studies, paving the way for advancements in understanding the oncobiome’s role in aging and neoplastic diseases.

## Therapeutic implications and future direction

The emerging understanding of oncobiome’s role opens new avenues for therapeutic interventions and highlights the potential for innovative approaches to treatment and prevention.

The identification of specific bacterial species associated with cancer progression presents a unique opportunity to explore antibiotics and antimicrobial strategies as potential therapeutic interventions. Targeted antibiotics could be employed to disrupt harmful bacterial populations, potentially slowing or reversing tumor growth. However, this approach requires careful consideration to avoid disrupting the beneficial components of the microbiome [179–181], unwanted drug-drug interaction during chemotherapy [182, 183], and to prevent the development of antibiotic resistance. Antimicrobial peptides (AMPs) represent another promising strategy, offering the possibility of selectively targeting cancer-associated bacteria with reduced risk of disturbing the overall microbiome balance [184].

Probiotics, live microorganisms that confer health benefits when administered in adequate amounts, and prebiotics, non-digestible fibers that promote the growth of beneficial bacteria, offer another strategy for modulating the microbiome in favor of cancer prevention and treatment [10, 185–190]. These interventions could help restore a healthy microbial balance, potentially reducing inflammation and inhibiting the growth of species with negative effects [185–188]. Clinical trials exploring the efficacy of specific probiotic strains and prebiotic compounds in modulating the microbiome and their impact on cancer outcomes are needed to validate this approach.

The field of oncobiome and oncobiosis is ripe with opportunities for groundbreaking research that could transform our understanding and treatment of the disease. Key future directions include leveraging detailed microbiome profiles to tailor prevention and treatment strategies to the individual’s unique microbial and genetic landscape, potentially improving the efficacy of therapies and reducing side effects. Further, investigating the potential for vaccines targeting specific species of the oncobiome associated with cancer may offer a proactive approach to prevention and treatment. Exploring the combination of microbiome-targeted therapies with conventional treatments like chemotherapy, radiation, and immunotherapy may lead to enhance overall treatment efficacy and potentially reduce side effects. Further research is also needed to elucidate the complex interactions between the microbiome/oncobiome, the immune system, and cancer cells, including the mechanisms by which bacteria influence cancer development and progression [3, 102, 103, 105, 191].

## Conclusion

This review aims at illuminating the pivotal role of oncobiosis, highlighting how microorganisms contribute to the disease’s initiation, progression, and possibly its response to treatment. The intricate interactions between the oncobiome and the host’s immune system, along with their influence on the tumor microenvironment, underscore the complexity of cancer’s etiology and the potential for microbial involvement in its pathology. Reflecting on the insights garnered, it becomes evident that the study of the oncobiome opens new avenues for the development of innovative diagnostics and therapeutic strategies. The identification of specific bacterial signatures associated with cancer offers the promise of novel biomarkers for early detection and prognosis, enhancing our ability to tackle the disease at its onset. Furthermore, understanding the mechanisms through which the oncobiome influences cancer initiation or progression provides a foundation for exploring antimicrobial treatments, probiotics, and prebiotics as potential adjuncts to conventional therapies, potentially improving outcomes and reducing side effects. However, the path to integrating microbiome research into breast cancer management is fraught with challenges, including the need for standardized methodologies in microbial analysis. Therefore, this review serves as a call to action for continued and expanded research in this emerging field. There is a pressing need for interdisciplinary collaboration among microbiologists, oncologists, geroscientists, and bioinformaticians to further elucidate the role of oncobiosis, refine diagnostic tools, and develop microbial-based interventions.

## Data Availability

Not applicable.
